# Dynamic diselenide bond‐enabled liquid crystal elastomer‐based two‐way shape memory aerogels with weldability and closed‐loop recyclability

**DOI:** 10.1002/smo.20230009

**Published:** 2023-10-12

**Authors:** Meng Wang, Jingshu Li, Hong Yang

**Affiliations:** ^1^ Institute of Advanced Materials School of Chemistry and Chemical Engineering State Key Laboratory of Digital Medical Engineering Southeast University Nanjing China

**Keywords:** aerogel, dynamic diselenide bond, liquid crystal elastomer, two‐way shape memory

## Abstract

Two‐way shape memory polymeric aerogels (2W‐SMPAs), with the ability to undergo reversible shape deformation in response to external stimuli, have extensive application in diverse fields such as actuators, sensors, robotics, and other relevant domains. In this study, we introduce a novel approach for fabricating a 2W‐SMPA material based on liquid crystal elastomers (LCEs) incorporating dynamic diselenide bonds. The aerogel exhibits liquid crystal phases, excellent compressibility and shape stability, and the mesogens are uniaxial‐oriented along the stretching direction. By capitalizing on the dynamic diselenide bonds, the LCE‐based aerogel demonstrated remarkable reprogrammability, weldability, and recyclability through thermal reorganization. The shape‐programmed aerogel sample exhibits reversible shrinking deformation during the heating and cooling cycles, ultimately achieving a maximum shrinkage ratio of 26.1%. Moreover, the LCE‐based aerogel's porous structure and monodomain orientation effectively enable the adsorption of the photothermal dye DR1 and facilitated the reversible photothermal‐induced shape deformation when exposed to 520 nm light irradiation. These findings reveal the potential application of this innovative LCE‐based aerogel material, enabled by dynamic diselenide bonds, in various areas including control devices, soft actuators, and other diverse fields.

## Graphical abstract

Employing thiol‐ene click chemistry and dynamic diselenide bonds, we prepared an LCE‐based two‐way shape memory aerogel possessing programmability, weldability and recyclability. The shape‐programmed aerogel exhibits reversible shrinking deformations under heat with a shrinkage rate of 26.1%. Leveraging the porous structure, the sample can effectively absorb DR1 and exhibit the reversible deformation under 520 nm light irradiation.

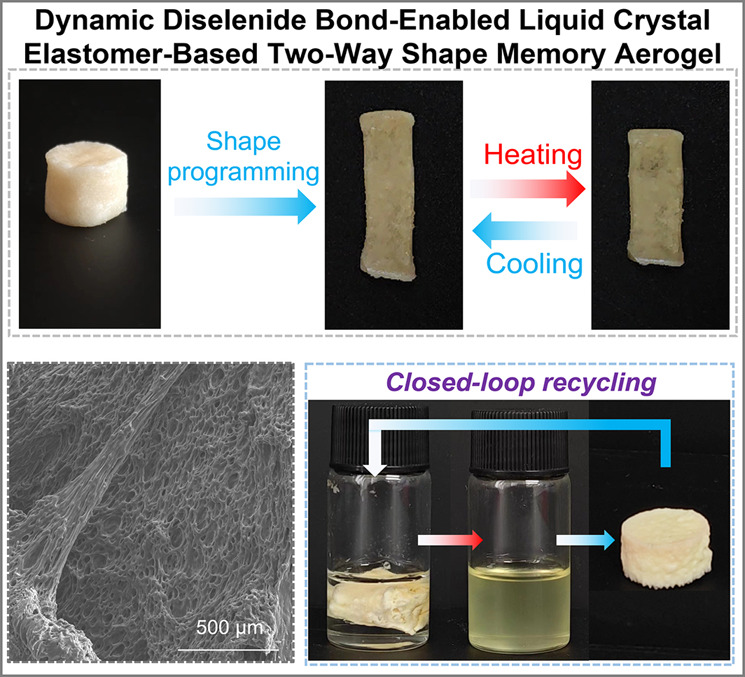

## 1 INTRODUCTION

Aerogels, first reported by Kistler in 1931, represent a new generation of solid porous open‐cell materials.^[1]^ These materials exhibit remarkable characteristics such as high porosity, low density, large specific surface area, low dielectric constant, and extremely low thermal conductivity.[^2^, ^3^, ^4^, ^5^, ^6^, ^7^] As a result, aerogels have been extensively investigated for their applications in photocatalysis, electrocatalysis, adsorption, separation, thermal insulation, and sensing.[^8^, ^9^, ^10^, ^11^, ^12^] Aerogels are typically fabricated through a three‐step sol‐gel process, which involves the use of precursor gels, aging, and drying, using organic materials, inorganic materials, organic‐inorganic hybrids, or other materials.[^13^, ^14^, ^15^] Among the various types of aerogels, shape‐memory polymeric aerogels (SMPAs) have garnered significant attention due to their unique ability to recover their original shapes in response to external stimuli.^[16]^ The first thermal‐responsive SMPA material with shape fixation and shape recovery capabilities was reported by Rowan in 2016.^[17]^ Since then, several novel SMPAs derived from traditional shape memory polymers have been developed. However, most of the previously reported SMPAs only exhibit one‐way shape memory behavior, transitioning from the temporary shape to the permanent shape. This limitation may restrict their potential applications in the field of actuators, sensors, robotics, etc. Therefore, the fabrication of SMPAs with the ability to undergo reversible shape‐morphing and possess porous structures presents new challenges in the field.

Designing and fabricating two‐way shape‐memory polymer aerogels (2W‐SMPAs) based on traditional two‐way shape‐memory polymers represents a feasible strategy. Liquid crystal elastomers (LCEs), as a fascinating category of such polymer materials, possess the combined characteristics of liquid crystals (LCs) with anisotropic properties and rubber elasticity.[^18^, ^19^, ^20^] LCEs have the capability to undergo large and reversible macroscopic shape deformations between two different permanent shapes, attributed to the change in molecular order of the mesogens during the LC‐to‐isotropic phase transition induced by external stimuli.[^21^, ^22^, ^23^, ^24^] Given their exceptional properties, LCEs hold great promise as the most suitable materials for the realization of novel 2W‐SMPAs.^[25]^ However, a paradox that cannot be ignored has arisen when introducing LCE into aerogel matrix. For the traditional fabrication process of LCEs, a pre‐crosslinked LCE with loosely crosslinked networks is initially formed within several hours, followed by shape programming and a second crosslinking step to further lock the anisotropic network. Indeed, the traditional process of preparing polymer aerogels is time‐consuming, typically spanning several days due to the completion of essential steps such as sol‐gel transition, solvent exchange, and drying.^[26]^ Consequently, this extended duration leads to the LCE‐based aerogel becoming fully cross‐linked before the shape programming phase, thereby hindering the achievement of bidirectional shape memory function.

In recent years, dynamic covalent bonds (DCBs) have been creatively incorporated into LCE materials, aiming to introduce rearrangement capabilities to polymer networks.[^27^, ^28^, ^29^, ^30^, ^31^, ^32^] The inclusion of DCBs enables LCE‐based materials to undergo exchange reactions under external stimuli, resulting in reshaping, reprocessing, and self‐healing capabilities that were previously absent in traditional LCEs. The pioneering work by Ji and colleagues has laid the foundation for significant advancements in the field of DCB‐based LCEs over the past years.[^28^, ^33^] Thus, designing and fabricating 2W‐SMPAs based on DCB‐containing LCE materials is a viable strategy. By incorporating DCBs, LCE‐based aerogels can acquire reprogrammable properties, which facilitate the shape programming process and mesogenic orientation of the aerogels. Considering the lengthy preparation process of aerogels, this is particularly advantageous as it ensures the formation of the aerogel framework to achieve good mechanical performance while allowing shape programming to achieve two‐way shape memory functionality. As a typical DCB, diselenide bonds possess a relatively low bond energy of approximately 172 kJ/mol and have been widely employed in the fabrication of LCE materials and soft actuators.^[34]^ Under mild conditions, the diselenide bond can undergo the selenium‐exchange reaction, which is dynamic in nature and can occur without the need for additional catalysts or auxiliary agents. Due to the distinctive properties of the diselenide bond, the LCE material possesses the ability to undergo shape programming, welding and recycling.

In this study, leveraging the utilization of diselenide bonds, we introduced DCBs into the LCE‐based aerogel materials, thereby simplifying the preparation process and achieving the development of novel 2W‐SMPAs. This method effectively overcame the challenge posed by prolonged preparation time in traditional aerogels, allowing for the shape programming of the LCE‐based aerogel after complete crosslinking, thereby achieving two‐way shape memory functionality. Initially, we employed the conventional polymer aerogel preparation procedure to synthesize a polydomain LCE‐based aerogel. The liquid crystal (LC) monomer was crosslinked with the diselenide‐containing chain extender using a thermally‐induced thiol‐ene click chemistry method, resulting in the formation of an organic gel. Then, a sol‐gel transition was induced, followed by supercritical CO_2_ drying technique, leading to the formation of a polydomain LCE‐based aerogel. Subsequently, the polydomain LCE‐based aerogel was subjected to a temperature of 120°C to trigger diselenide bond exchange, followed by uniaxial stretching to induce the alignment of mesogens along the longitudinal axis. Upon cooling back to room temperature, an oriented LCE‐based aerogel was obtained. The mesomorphic properties, porous structures, mechanical characteristics, weldable and closed‐loop recyclable abilities, two‐way shape‐memory behavior, and practical applications of the prepared LCE‐based aerogel were comprehensively investigated in this study.

## 2 RESULTS AND DISCUSSION

**FIGURE 1:**
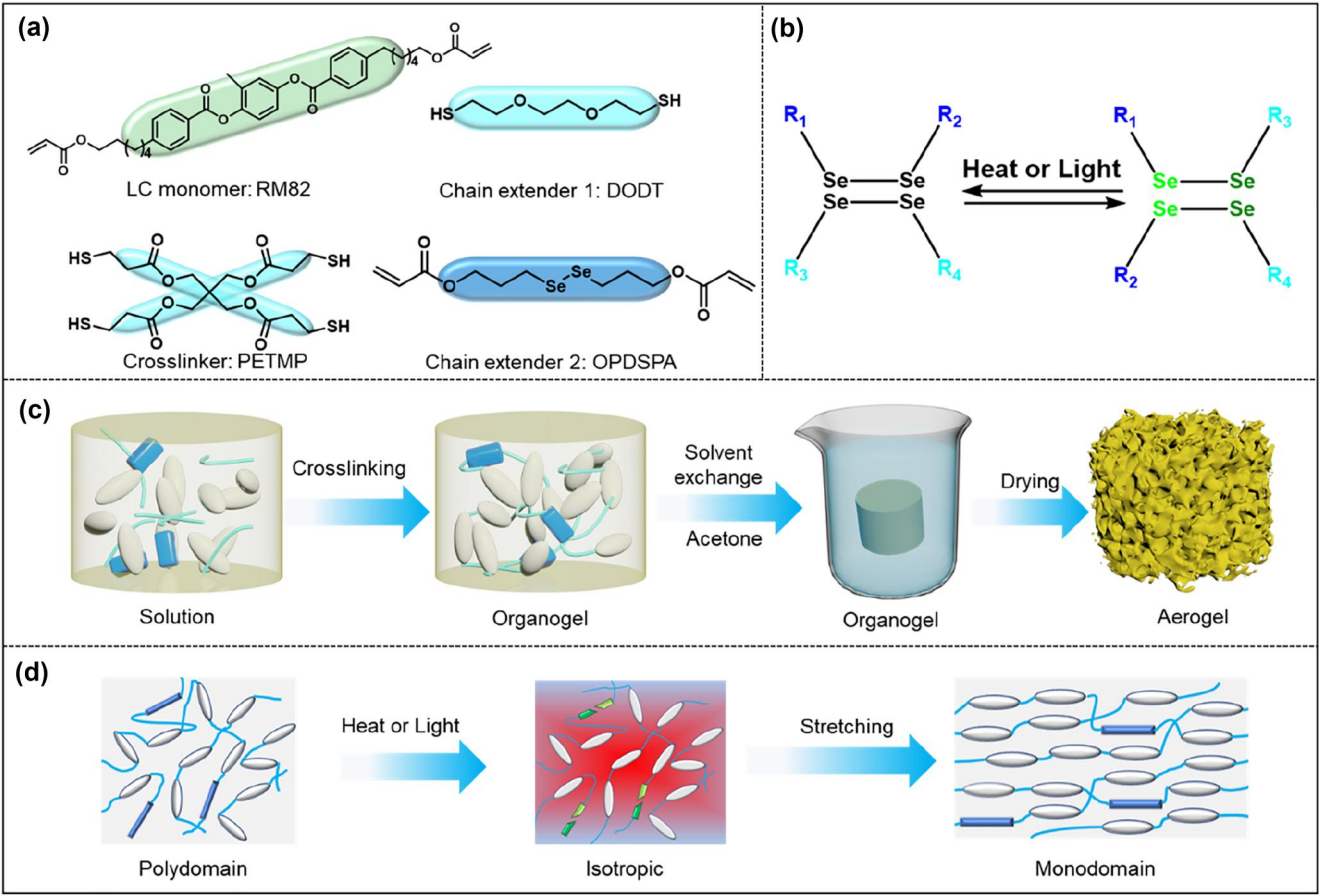
(a) Chemical structures of RM82, DODT, PETMP and OPDSPA. (b) Schematic illustration of the diselenide bond exchange. (c), (d) Schematic diagrams of the fabrication process of LCE‐based aerogels.

The chemical constituents employed in the preparation of LCE‐based aerogel containing diselenide bonds are depicted in Figure 1a. Previous studies have demonstrated that the exchange reaction of diselenide bonds can be triggered by either a specific temperature or visible light irradiation.^[33]^ As illustrated in Figure 1b, under external stimulation, the diselenide bonds undergo cleavage, while upon the removal of the stimulus, new diselenide bonds reform. Capitalizing on this characteristic, we selected the diselenide‐containing chain extender diselanediylbis(propane‐3,1‐diyl) diacrylate (OPDSPA) to confer rearrangeable networks to the LCE‐based aerogel. The synthetic procedure for OPDSPA is displayed in Figure S1. The ^1^H NMR and high‐resolution mass spectra of the intermediate are shown in Figure S2 and S3, respectively. The ^1^H NMR spectrum of OPDSPA is exhibited in Figure S4. Traditional sol‐gel process and supercritical drying method were employed to prepare the LCE‐based aerogel. A 10 mL transparent glass bottle was utilized as the mold for the aerogel preparation. The schematic depiction of the preparation procedures for the polydomain LCE‐based aerogel is presented in Figure 1c, Figure S5 and detailed in the Supporting Information. In summary, all the materials were combined in a container, and the organic gel was prepared through the thermal‐induced thiol‐ene click reaction. Following aging, solvent exchange, and supercritical CO_2_ drying, the LCE‐based aerogel fabrication process was completed. To obtain a monodomain LCE‐based aerogel with two‐way shape‐memory functionality, as illustrated in Figure 1d, the initial samples in the polydomain state were annealed at 120°C for 40 min to induce diselenide bond cleavage, resulting in an isotropic state. Subsequently, the samples were stretched to 250% of their original length, fixed, cooled to room temperature, and left undisturbed for 24 h to obtain the monodomain LCE‐based aerogel sample.

The thermal stability of the LCE‐based aerogel incorporating diselenide bonds was investigated using a thermal gravimetric analyzer (TGA), and the corresponding results are presented in Figure S6. Under a nitrogen atmosphere, negligible mass loss was observed in the LCE‐based aerogel sample from room temperature up to 310°C. At 310°C, the sample exhibited a 5% mass reduction, indicating an initial thermal decomposition. Subsequently, between 310°C and 450°C, the rate of mass loss in the LCE‐based aerogel sample increased, suggesting continuous breakdown and collapse of the internal skeleton. Beyond 450°C, the mass loss of the sample plateaued, indicating the completion of the thermal decomposition process. Overall, the LCE‐based aerogel sample demonstrated favorable thermal stability.

**FIGURE 2:**
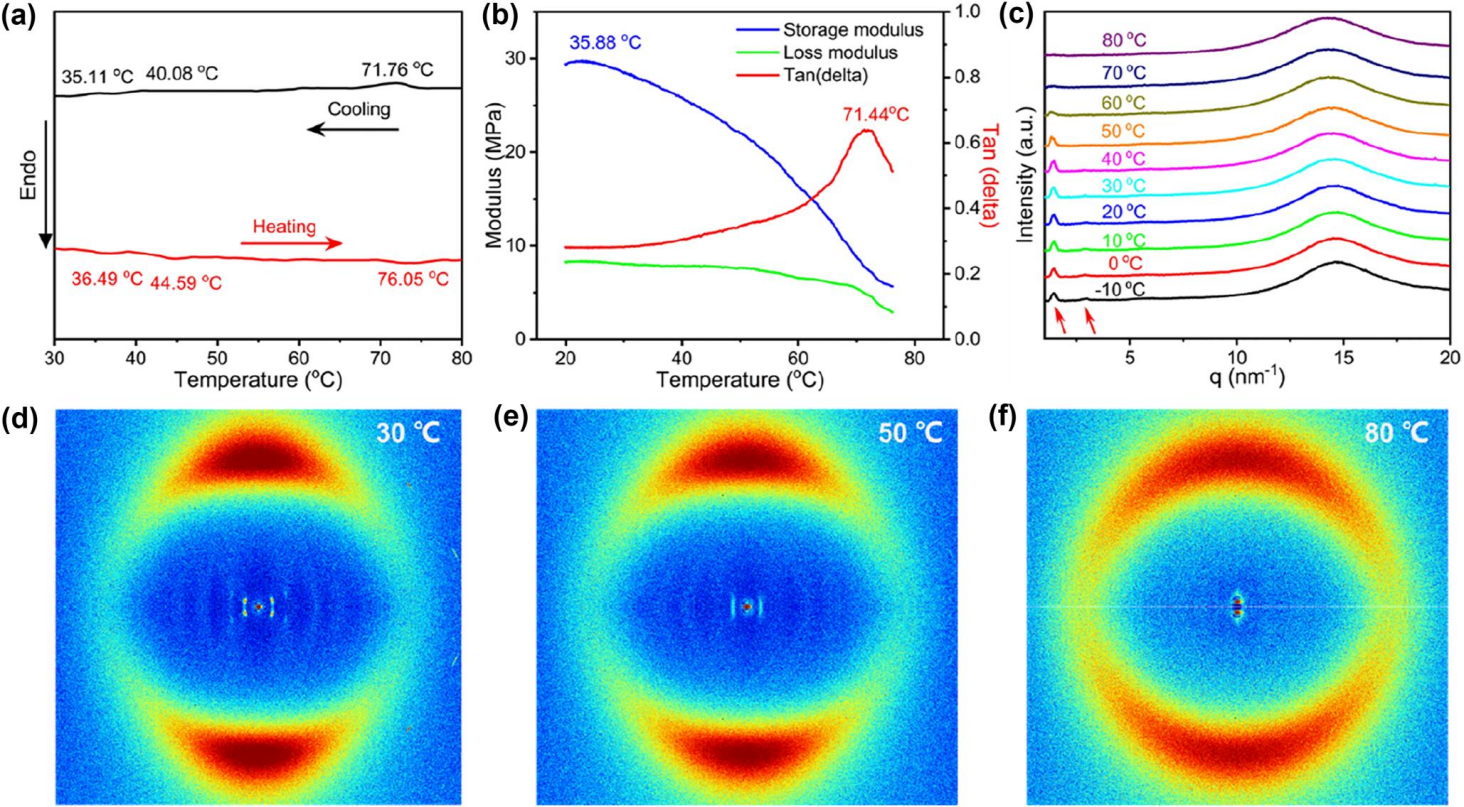
(a) DSC curves of the LCE‐based aerogel sample during the first cooling and second heating scans at a rate of 10°C min^−1^ under nitrogen atmosphere. (b) Oscillation‐temperature curve of the LCE‐based aerogel sample. (c) 1D‐WAXS and (d)‐(f) 2D‐WAXS patterns of the LCE‐based aerogel sample during the heating process.

To further explore the thermal and mesomorphic characteristics of the LCE‐based aerogel, differential scanning calorimetry (DSC) was employed. The resulting DSC curve of the LCE‐based aerogel sample is presented in Figure 2a. Three temperature points were recorded during the heating process, namely 36.49°C, 44.59°C, and 76.05°C. Similarly, during the cooling process, three temperature points were identified at 35.11°C, 40.08°C, and 71.76°C, respectively. Based on these observations, it was initially inferred that the LCE‐based aerogel containing diselenide bonds possessed mesomorphic phases. To verify this inference, we conducted the oscillation‐temperature ramp tests using a dynamic mechanical analyzer (DMA). As illustrated in Figure 2b, with the increase in temperature, the storage modulus (E′) demonstrated a gradual decline, commencing at 35.88°C, thereby suggesting the presence of glass phase transition phenomena within the investigated temperature range. The dissipation factor (tanδ) exhibited a pronounced peak at 71.44°C, indicating the presence of a phase transition process in the sample. These two sets of data correspond to the phase transition points observed in the DSC analysis, specifically the glass transition temperature (T_g_) at approximately 35.88°C and the LC‐to‐isotropic phase transition temperature at around 71.44°C.

One‐dimensional (1D) and two‐dimensional (2D) wide‐angle X‐ray scattering (WAXS) techniques were employed to investigate the LC phases and orientation of mesogens in LCE‐based aerogel samples. The temperature range for the testing process was −10°C–80°C, as illustrated in Figure 2c. Analysis of the 1D‐WAXS plot revealed the presence of two small peaks in the small‐angle region. The more prominent peak was observed at *q* = 1.41 nm^−1^, gradually diminishing with increasing temperature until it completely disappeared at 80°C. Another weak peak was located at *q* = 2.84 nm^−1^, which vanished after heating up to 50°C. The ratio of *q* values between these two peaks was found to be 1:2. In the wide‐angle region, a diffuse envelope peak (*q* = 14.46 nm^−1^) persisted throughout the testing process. Based on the previous findings from DSC and temperature ramp tests, it was inferred that within the temperature range of 36.49°C–44.59°C, the material exhibited characteristics of a smectic phase. As the temperature continued to rise within the range of 44.59°C to 76.05°C, the material exhibited properties characteristic of the nematic phase. Beyond 76.05°C, the LCE‐based aerogel underwent a transition into the isotropic state. Furthermore, as illustrated in Figure 2d, the 2D‐WAXS measurements depicted the presence of a quadruple scattering halo in the small‐angle region and a pair of crescents in the wide‐angle region at 30°C, indicating the characteristic features of a smectic C phase and well‐aligned mesogens. Subsequent heating to 50°C led to the disappearance of the scattering peak at a *d*‐spacing of 2.21 nm, while the peak corresponding to a *d*‐spacing of 4.45 nm gradually diminished, signaling the emergence of nematic phase characteristics. As the temperature approached approximately 80°C, the crescents in the wide‐angle region transformed into a diffuse ring, indicating the transition of the LCE‐based aerogel sample into the isotropic state. The order parameter *S* of the LCE‐based aerogel sample at different temperatures was calculated using the following equation to evaluate the extent of orientation of mesogens.[^35^, ^36^]

$$S=\frac{1}{2}(3\langle \text{co}s^{2}\alpha \rangle -1)$$

$$\langle \text{co}s^{2}\alpha \rangle =\frac{\int_{0}^{\pi }I\left(\alpha \right)\left|\sin\;\alpha \right|\text{co}s^{2}\alpha d\alpha }{\int_{0}^{\pi }I\left(\alpha \right)\left|\sin\;\alpha \right|d\alpha }$$

I(α) represented the intensity profiles integrated in azimuthal angle *α*, according to the 2D‐WAXS patterns. At 30°C, the sample exhibited an *S* value of approximately 0.64, which signified the uniaxial orientation of the mesogens along the stretching direction. With increasing temperature, the *S* value of the sample exhibited a gradual decline, reaching 0.48 at 50°C and then further decreasing to 0.12 at 80°C. This trend indicated the transition from the LC phase to isotropic phase of the LCE‐based aerogel sample.

**FIGURE 3:**
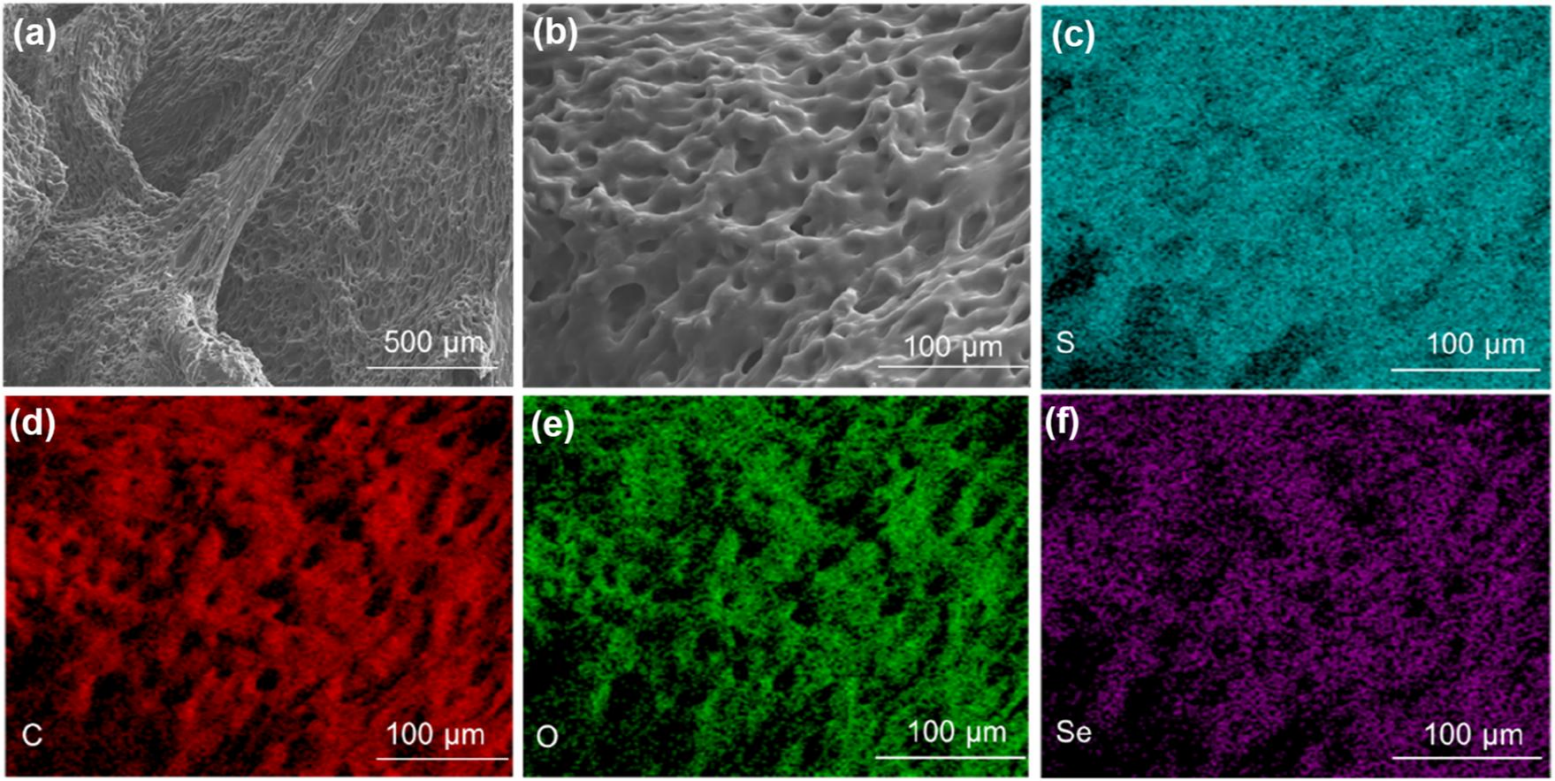
(a), (b) SEM images of the surface of LCE‐based aerogel samples. (c)‐(f) Distribution maps of four elements on the surface layer of the LCE‐based aerogel sample.

The internal surface morphology and porous structure of the LCE‐based aerogel sample were examined using scanning electron microscopy (SEM). As shown in Figure 3 and Figure S7, the sample displayed a distinct porous structure uniformly distributed on the surface, without any noticeable pore wall wrinkles. This observation indicates that the introduction of a dynamic crosslinked polymer network based on diselenide bonds into the aerogel did not cause the collapse of the porous structure of the aerogel sample during aging, solvent exchange, and supercritical CO_2_ drying processes. Furthermore, Figures 3c‐3f and Table S1 illustrate that the four different elements (C, O, S, Se) were uniformly distributed within the aerogel matrix. In addition, we utilized the mercury intrusion porosimetry (MIP) technique to obtain information regarding the pore volume corresponding to each pore size. Figure S8 depicts the pore size distribution density function, representing the overall range of pore sizes divided into several (or infinite) pores with a unit increment of 1 nm. If a pore exists at a specific nanoscale position, its pore volume is represented on the vertical axis. It could be observed that there were pore structures of varying sizes ranging from 5 to 500 nm and its average aperture was 119.51 ± 13.20 nm. Furthermore, we employed an automatic true density analyzer to measure the volume density and skeletal density of the sample. The measured bulk density (ρ_b_) of the sample was 0.31 ± 0.01 g·cm^−3^, while the skeletal density (ρ_s_) was determined to be 1.15 ± 0.03 g·cm^−3^. By calculating the sample porosity using the formula (1 − ρ_b_/ρ_s_) × 100%, the porosity of the sample at room temperature was estimated to be approximately 73%, indicating the typical porous structure exhibited by the LCE‐based aerogel sample.

**FIGURE 4:**
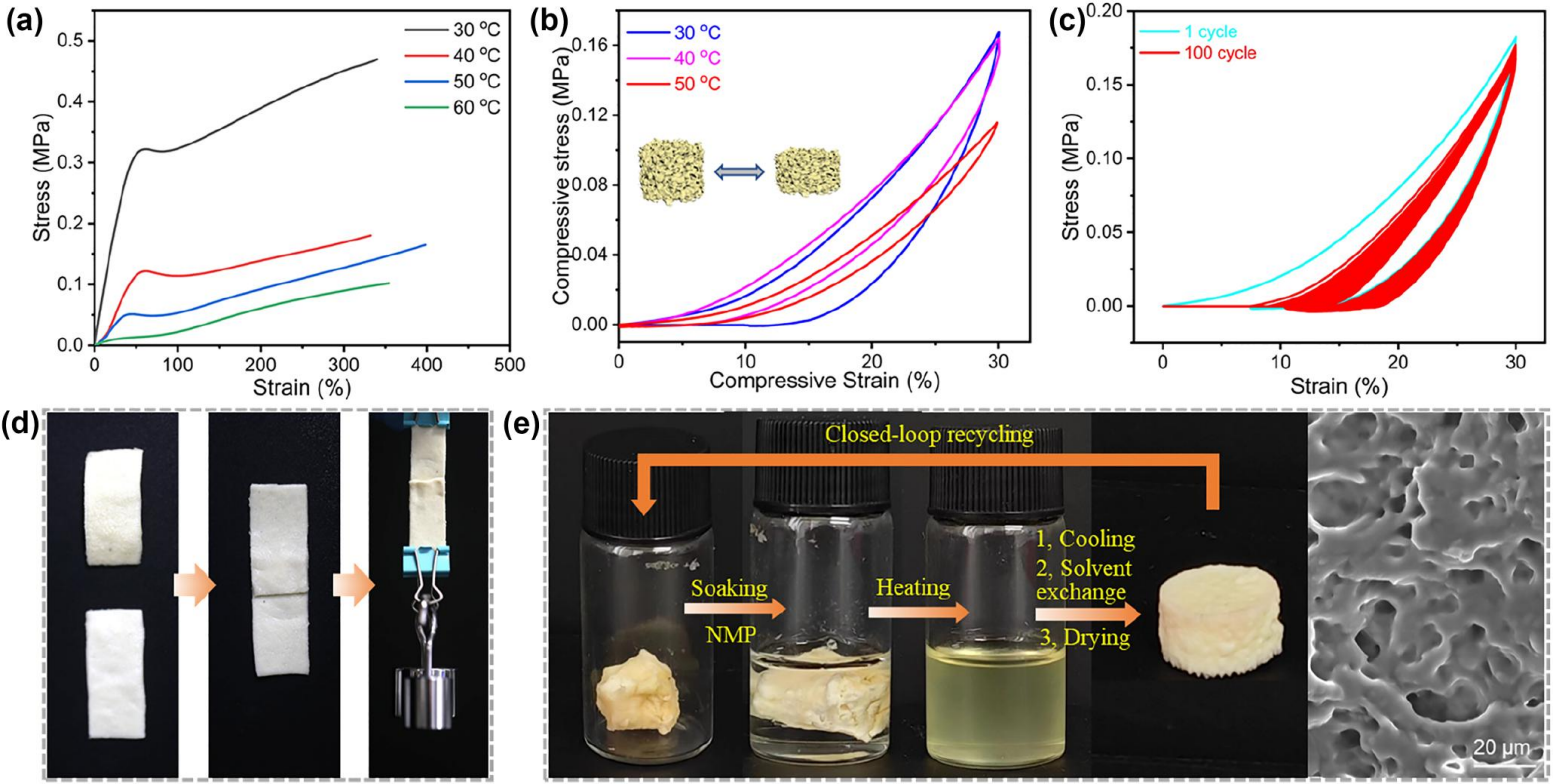
(a) Tensile and (b) compressive stress‐strain curves of LCE‐based aerogel samples at different temperatures. (c) Stress‐strain curves of the LCE‐based aerogel sample during 100 compression cycles at 30°C. (d) An assembled LCE‐based aerogel sample by welding two monoliths together. (e) The closed‐loop recycling of the LCE‐based aerogel samples and SEM image of the new‐formed sample.

To investigate the mechanical properties of LCE‐based samples, the influence of temperature on the tensile properties of the LCE‐based sample was first examined using DMA, as depicted in Figure 4a. At 30°C, the sample exhibited an elastic modulus of 1.05 ± 0.08 MPa, a tensile strength of 0.46 ± 0.03 MPa, and a fracture elongation of 327 ± 23%. As the temperature increased, the mechanical performance gradually decreased, with the elastic modulus showing the most significant decrease. However, the fracture elongation remained around 281% after reaching 40°C. Furtherly, compression‐decompression tests were conducted on LCE‐based samples using DMA compression molds. The results, presented in Figure 4b, reveal the behavior of the samples at different temperatures (30°C, 40°C, and 50°C) after applying a 30% compressive strain in the vertical direction. The samples exhibited rapid recovery to their original shape without significant damage and demonstrated notable flexibility. As the temperature continued to rise, the compressive stress of the sample at a strain of 30% exhibited a significant decrease at 50°C due to the glass phase transition and reduction of the mesogenic order. Additionally, Figure 4c displays the stress‐strain curve of the sample subjected to 100 cycles of compression‐decompression at a 30% compression ratio at room temperature. After 100 cycles, there was no significant attenuation in the maximum compressive stress at a 30% strain, indicating the excellent compressibility and shape stability of the LCE‐based aerogel sample. As reported in our previous work, the diselenide exchange reaction can occur at moderate temperatures without the need for any additional agents, and higher temperatures can promote a faster and more efficient exchange reaction.^[34]^ The stress relaxation curves for the aerogel at different temperatures were measured using DMA and depicted in Figure S9. As the temperature increased, the relaxation time decreased, signifying a more rapid exchange of diselenide bonds. Notably, beyond 90°C, there was a significant enhancement in the rate of diselenide bond cleavage and reformation. Based on these data, we selected a temperature of 120°C for the welding and recycling of the aerogels.

To further investigate the weldable and recyclable capabilities of the LCE‐based aerogel samples, two aerogel samples, each weighing 0.70 g, were overlapped at a temperature of 120°C, and a weight of 50 g was applied to the overlapping region to apply pressure. Following a 40‐min period of hot welding without the use of any auxiliary reagents, an assembled aerogel sample was obtained, and the SEM image of the overlap part showed no discernible boundary (Figure S10). As illustrated in Figure 4d, the assembled aerogel demonstrated good mechanical performance by successfully suspending a weight of 150 g without experiencing any breakage. At 30°C, the welded LCE‐based aerogel sample exhibited an elastic modulus of 0.92 ± 0.05 MPa, a tensile strength of 0.18 ± 0.01 MPa, and a fracture elongation of 200 ± 17%, as detailed in Figure S11. In comparison to the monolithic LCE‐based aerogel sample, the welded aerogel sample exhibited a decrease in mechanical properties. This decrease could be attributed to the inevitable presence of structural defects at the welded interface of the porous aerogel samples, in contrast to the smooth surface of traditional LCE films. Further, we conducted a closed‐loop recycling experiment on the LCE‐based aerogel sample, as shown in Figure 4e. The 0.70 g sample was soaked in a 3 mL solution of N‐methyl‐2‐pyrrolidone (NMP) (boiling point 202°C), and no dissolution was observed. After heating at 120°C for 1 h, the sample gradually dissolved due to the cleavage of diselenide bonds, resulting in a clear solution. After cooling back to room temperature overnight, the diselenide bond reformed and the dissolved solution underwent gelation, resulting in the formation of an organic gel. Subsequently, the gel was subjected to aging, solvent exchange, and supercritical CO_2_ drying steps, leading to the preparation of a new sample. SEM analysis revealed the presence of porous structure within the sample, while automated true density analyzer testing indicated a skeletal density of 1.14 ± 0.03 g/mL and a bulk density of 0.33 ± 0.01 g/mL at room temperature, resulting in a calculated sample porosity of approximately 71%. In summary, these experimental results indicated the successful closed‐loop recycling of LCE‐based aerogels.

**FIGURE 5:**
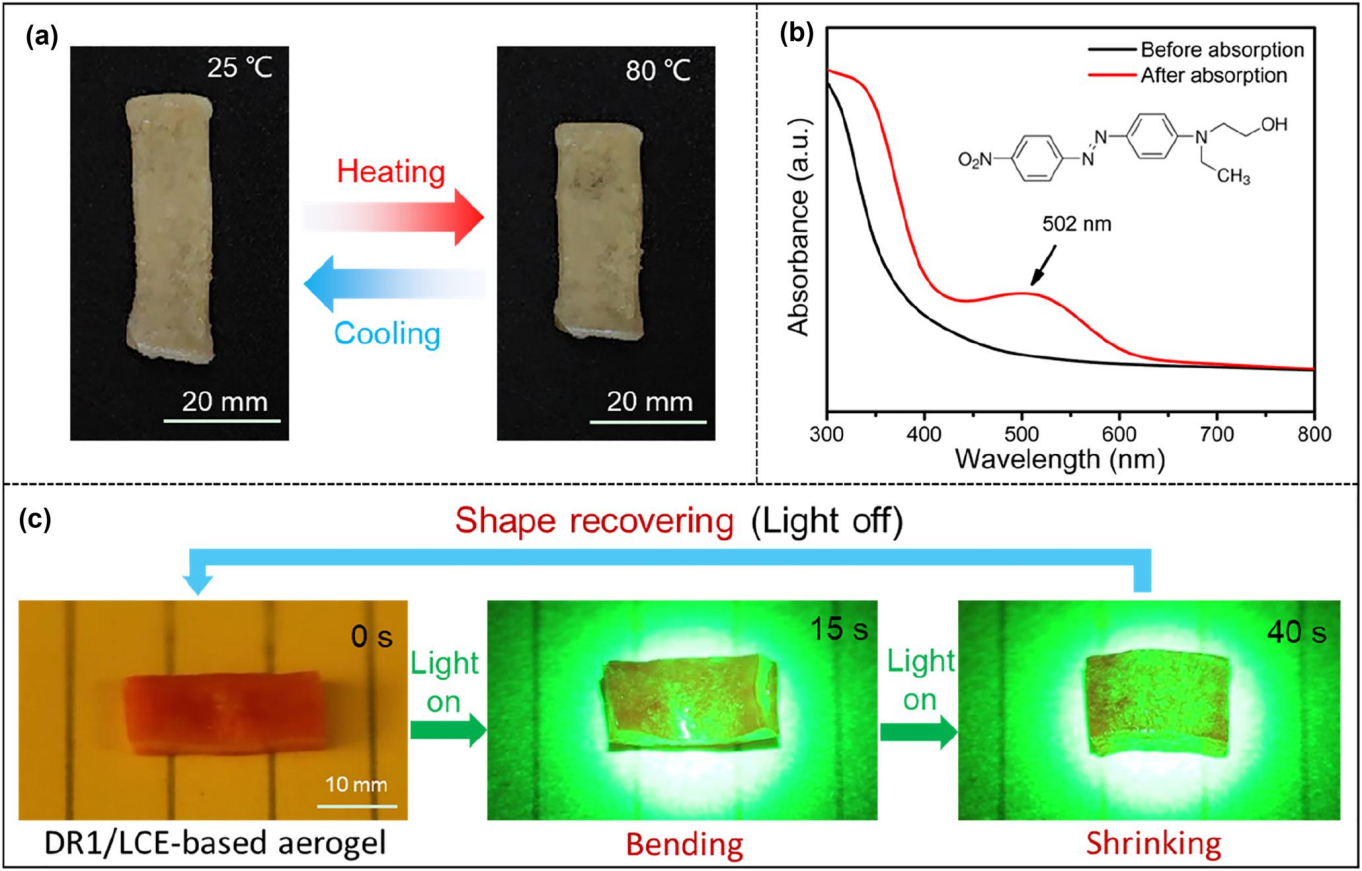
(a) Thermal‐induced two‐way shape memory behavior of the LCE‐based aerogel sample. (b) UV‐vis absorption spectra of the LCE‐based aerogel sample before and after adsorbing DR1. (c) Light‐driven shape deformation of the DR1/LCE‐based aerogel sample under green light illumination (wavelength: 520 nm, intensity: 200 mW/cm^2^).

As mentioned in the Introduction section, the reversible deformation ability of the LCE‐based 2W‐SMPA material is the most desired and attractive property in this work. The monodomain LCE‐based aerogel sample was placed on a hot stage and heated. As shown in Figure 5a and Video S1, as the temperature increased, the sample exhibited reversible shrinking deformation along the stretching direction. When the temperature reached above the clearing point temperature at 78°C, the sample eventually contracted to a minimum length of approximately 3.37 cm. After turning off the heating, as the temperature decreased, the sample gradually recovered its length. Upon reaching room temperature, the sample length recovered to approximately 4.25 cm. This reversible contraction/extension deformation can be repeatedly achieved through repeated heating/cooling cycles. During the heating‐cooling process, the sample exhibited a maximum shrinkage rate, defined as (L_0_ − L_iso_)/L_iso_, of 26.1%, where L_iso_ represents the minimum length of the sample in the isotropic phase and L_0_ is the longitudinal length of the LCE‐based aerogel measured at room temperature. DMA was further used to investigate the shape recovery of this LCE‐based aerogel with the change of temperature. As shown in Figure S12, under the isoforce mode, the contraction strain of the LCE‐based aerogel sample, represented by (L − L_0_)/L_0_ (where L is the measured length of the LCE‐based aerogel sample at a given temperature), varied from 0% to −20.7% during the heating process from 30 to 80°C. Upon cooling, the strain of the sample returned to 0, indicating complete recovery. Furthermore, the strain of the LCE‐based aerogel exhibited reversible changes during subsequent heating and cooling cycles, indicating the excellent reversibility and repeatability of the material.

Conventional functional LCEs typically introduced functional fillers into the system through doping or swelling effects to form composite materials, often leading to compromised mechanical properties of the LCEs. As a porous material, LCE‐based aerogels have adsorption capabilities, allowing direct adsorption of functional fillers without compromising the inherent properties of the material. Herein, we explored the adsorption capability of LCE‐based aerogel samples on various organic dyes, including disperse red 1(DR1), methylene blue, Rhodamine B, and Rhodamine 700. As depicted in Figure S13, different dyes were dissolved in anhydrous ethanol to create a composite solution with the same concentration of 10 mg/L. Subsequently, the aerogels were immersed in the solution, and the ultraviolet‐visible (UV‐vis) absorption spectra of the composite solution were measured before and after a 4‐h adsorption period. The dyes/ethanol solutions all exhibited significant attenuation in their corresponding wavelength ranges in the UV‐vis absorption spectra after the adsorption process. Furthermore, it is worth noting that there were no observed changes in the shape of the LCE‐based aerogel samples during the adsorption process. This suggests that the adsorption capability of the aerogel samples toward the dyes is primarily attributed to their porous structure, rather than the conventional swelling effect of traditional LCE materials in solvents.

We selected DR1 as a representative dye and conducted tests to evaluate the photo‐responsive properties of the aerogel sample after adsorbing DR1. The monodomain LCE‐based aerogel samples were immersed in an ethanol solution containing DR1 (10 mg/L) for a period of 4 h. After carefully removing excess solvent from the surface of the samples and allowing them to air dry, the aerogel sample adsorbed with DR1 was obtained. As depicted in Figure 5b, the DR1/LCE‐based aerogel sample exhibited a conspicuous absorption peak at the wavelength of 502 nm in the UV‐vis absorption spectra. Before conducting the light‐driven actuation, stress relaxation tests were performed on the pristine aerogel without dye absorption, under various intensities of 520 nm light. As shown in Figure S14, with the light intensity increasing from 0 to 300 mW/cm^2^, the relaxation time gradually decreased. Even under light illumination with an intensity of 300 mW/cm^2^, the characteristic relaxation time, defined as the time required to reach 37% (1/e) of the normalized relaxation modulus, remained longer than 500 s. This observation indicated that the influence of diselenide exchange under light illumination could be disregarded during the subsequent rapid photothermal‐driven actuation of the DR1/LCE‐based aerogel.

The free‐standing DR1/LCE‐based aerogel sample was exposed to green light (wavelength: 520 nm, intensity: 200 mW/cm^2^) in order to investigate the light‐driven shape memory behavior. As depicted in Figure 5c and demonstrated in Video S2, the deformation process of the sample under green light consisted of two distinct stages: bending and shrinking. Initially, the sample underwent bending deformation within the first 15 s, followed by a subsequent contraction along the stretching direction over the next 25 s. The maximum deformation of the sample was reached within 40 s, with the length reducing from 2.30 to 1.86 cm. This shape deformation sequence demonstrated the presence of a photo‐absorption gradient in the aerogel sample, considering the thickness of aerogel sample. Upon light absorption, the photothermal conversion led to a quick temperature rise in the surface, inducing the contraction deformation of the surface. However, the underlying layers did not experience a temperature increase, creating asymmetric internal stresses that resulted in the macroscopic bending deformation of the sample in the first 15 s. As the duration of light exposure extended, the overall temperature of the sample increased, leading to a comprehensive shrinking deformation in the next 25 s. Upon removal of the light stimulation, the sample exhibited expansion deformation and gradually recovered to the original state in 90 s. DMA was employed to further investigate the shape recovery of this LCE‐based aerogel under light illumination. As illustrated in Figure S15, under the isoforce mode, the LCE‐based aerogel sample exhibited a contraction strain ranging from 0% to −19.1% when exposed to light (wavelength: 520 nm, intensity: 200 mW/cm^2^) for 40 s. Upon deactivating the light source, the strain of the sample reverted to 0 within 90 s, signifying complete recovery. Additionally, the DR1/LCE‐based aerogel demonstrated reversible changes in strain during consecutive cycles of green light exposure, exhibiting excellent reversibility and repeatability.

## 3 CONCLUSION

In summary, employing thiol‐ene click chemistry and dynamic diselenide bonds, we have successfully synthesized an LCE‐based aerogel sample with two‐way shape memory capabilities, utilizing conventional sol‐gel methodology and supercritical CO_2_ drying processes. Based on the comprehensive characterization results, it is evident that the diselenide bonds incorporated LCE‐based aerogel exhibited both the liquid crystal phases, good mechanical properties and well‐defined porous structures, and the inside mesogens were uniaxial‐oriented along the stretching direction. Benefiting from the presence of dynamic diselenide bonds, the LCE‐based aerogel materials exhibited remarkable reprogrammability, weldability, and recyclability, allowing for the attainment of monodomain‐oriented LCE‐based aerogels through thermal reorganization. These novel aerogel materials demonstrated reversible shrinking deformations under thermal stimuli, with a maximum shrinkage rate of 26.1%. Notably, after multiple cycles, no significant attenuation was observed. Leveraging the porous structure, the aerogel samples exhibited effective adsorption of various photothermal dyes without compromising material integrity. Furthermore, we have successfully demonstrated the reversible photothermal‐induced deformation behavior of the LCE‐based aerogel, absorbed with DR1, under 520 nm light irradiation. These findings broaden the application prospects of shape memory aerogels in the field of photo‐induced deformable materials.

## CONFLICT OF INTEREST STATEMENT

The authors declare no conflicts of interest.

## ETHICS STATEMENT

No animal or human experiments were involved in this study.

## Supporting information

Supporting Information S1

Video S1

Video S2

## Data Availability

The data that support the findings of this study are available in the supplementary material of this article.
