# Supplementary material for: Dynamic diselenide bond‐enabled liquid crystal elastomer‐based two‐way shape memory aerogels with weldability and closed‐loop recyclability
Source: Smart Mol. 2023 Oct 12;1(3):e20230009. doi: 10.1002/smo.20230009 (PMC12118233; doi:10.1002/smo.20230009)
Supplement: Supplementary file 1 — Supporting Information S1 [file SMO2-1-e20230009-s002.docx]

Supporting Information

Dynamic Diselenide Bond-Enabled Liquid Crystal Elastomer-Based Two-Way Shape Memory Aerogels with Weldability and Closed-Loop Recyclability

Meng Wang, Jingshu Li and Hong Yang*

**Materials and methods.**

LC monomer 1,4-bis-[4-(6-acryloyloxyhexyloxy) benzoyloxy]-2-methyl benzene (RM82, 98%) was purchased from Shijiazhuang Yesheng Chemical Technology Co., Ltd. 2,2'-(Ethane-1,2-diylbis(oxy)) diethanethiol (DODT) (99%) and 3-bromopropan-1-ol were purchased from TCI (Shanghai). Selenium, sodium borohydride (NaBH_4_), pentaerythritol tetra(3-mercaptopropionate) (PETMP), acryloyl chloride and dipropylamine were purchased from Energy Chemical. Triethylamine and MgSO_4_ were purchased from Sinnopharm Chemical Reagent Co., Ltd. All solvents were dried and distilled according to standard procedures. Other reagents were used without further purification.

All ^1^H NMR spectra were recorded on a Bruker HW600 MHz spectrometer (AVANCE AV-600) using CDCl_3_ as the solvent and δ 7.26 (CDCl_3_) as the internal reference. High-resolution mass spectrum was obtained through Thermo Scientific Hybrid Quadrupole-Orbitrap mass spectrometer in negative ion mode. Differential scanning calorimetry (DSC) thermographs were recorded on a TA Instruments Q2000 instrument (New Castle, DE) under nitrogen purge with a heating/cooling rate of 10 °C/min. Both one-dimensional (1D) WAXS and two-dimensional (2D) WAXD experiments were performed on Anton Paar SAXS point 2.0 with a TCS tage 300 temperature controller. The specimen (in a TCS sample holder) was placed in the sample chamber, which was evacuated to a pressure below 3 mbar in order to minimize the atmospheric scattering of the X-ray beam. For each specimen, six frames of 900-s exposures were collected and averaged. Two-dimensional data was transformed to one-dimensional curves by using SAXS analysis software (Anton Paar). All mechanical property studies of the polymeric samples were performed on a dynamic mechanical analyzer (DMA Q850, TA Instrument) using tension clamp. All the experiments were performed in triplicate. The oscillation-temperature ramp tests were conducted within the temperature range of 20 °C to 80 °C. These tests were performed under a preload force of 0.01 N, employing a heating rate of 3 °C/min, an amplitude of 20.0 μm, and a frequency of 1.0 Hz. In the quasi-static stress-strain experiments, the strain rate was set as 0.1 N/min. In the isoforce experiments for the thermally actuated samples, the samples were heated and cooled between 30 °C and 80 °C under a preload force of 0.001 N at a rate of 10 °C/min. In the isoforce experiments involving the light-actuated samples, the specimens were exposed to 520 nm light (200 mW/cm^2^, with an an "on" period of 40 s and an "off" period of 100 s) while under a preload force of 0.001 N. Stress relaxation experiments were conducted on the samples using a 5% strain at designated temperatures or light intensities. The SEM images were recorded on an Inspect F50 S3 field emission scanning electron microscope (FEI-SEM, America). A MW-GX-520 green light (Changchun Laser Optoelectronics Technology Co., Ltd., China) was used to irradiate on the samples. The light intensities of the lamp illuminating at different distances were accurately measured by using an optic power meter (LP-3B, Beijing Wuke Photoelectric Technique Co., Ltd., China).

**Synthesis of diselanediylbis(propane-3,1-diyl) diacrylate (OPDSPA)**


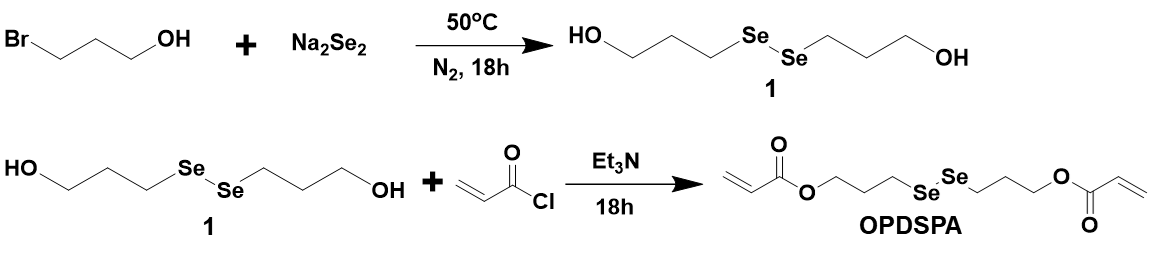


**Figure S1.** Synthetic route of chain extender OPDSPA.

A three-necked flask (250 mL) was charged with selenium powder (4.00 g, 50.64 mmol), sodium borohydride (1.92 g, 50.64 mmol), and deionized water (80 mL) under a nitrogen atmosphere. The mixture was heated to 50°C for 30 minutes. Subsequently, 3-bromo-1-propanol (3.52 g, 25.32 mmol) was dissolved in 80 mL of tetrahydrofuran and added to the reaction solution in the three-necked flask (250 mL) via a syringe. The reaction was allowed to proceed for 18 hours. The tetrahydrofuran solvent was then removed using vacuum rotary evaporation. The remaining solution was extracted three times with dichloromethane and purified using saturated salt water three times. The organic phases were combined, dried with anhydrous sodium sulfate, and filtered. The crude product was further purified through a fast chromatographic column using a dichloromethane and methanol mixture (DCM:MeOH = 20:1) as the eluent. The compound **1** obtained was a yellow oil (2.10 g, yield: 60.21%). ^1^H NMR (600 MHz, CDCl_3_) δ: 3.75-3.73 (t, *J* =12 Hz, 4H), 3.03-3.01 (t, *J* =12 Hz, 2H), 2.16 (s, 2H), 2.02-1.98 (m, 4H). HRMS m/z: 276.9245 [m-H]^−^.


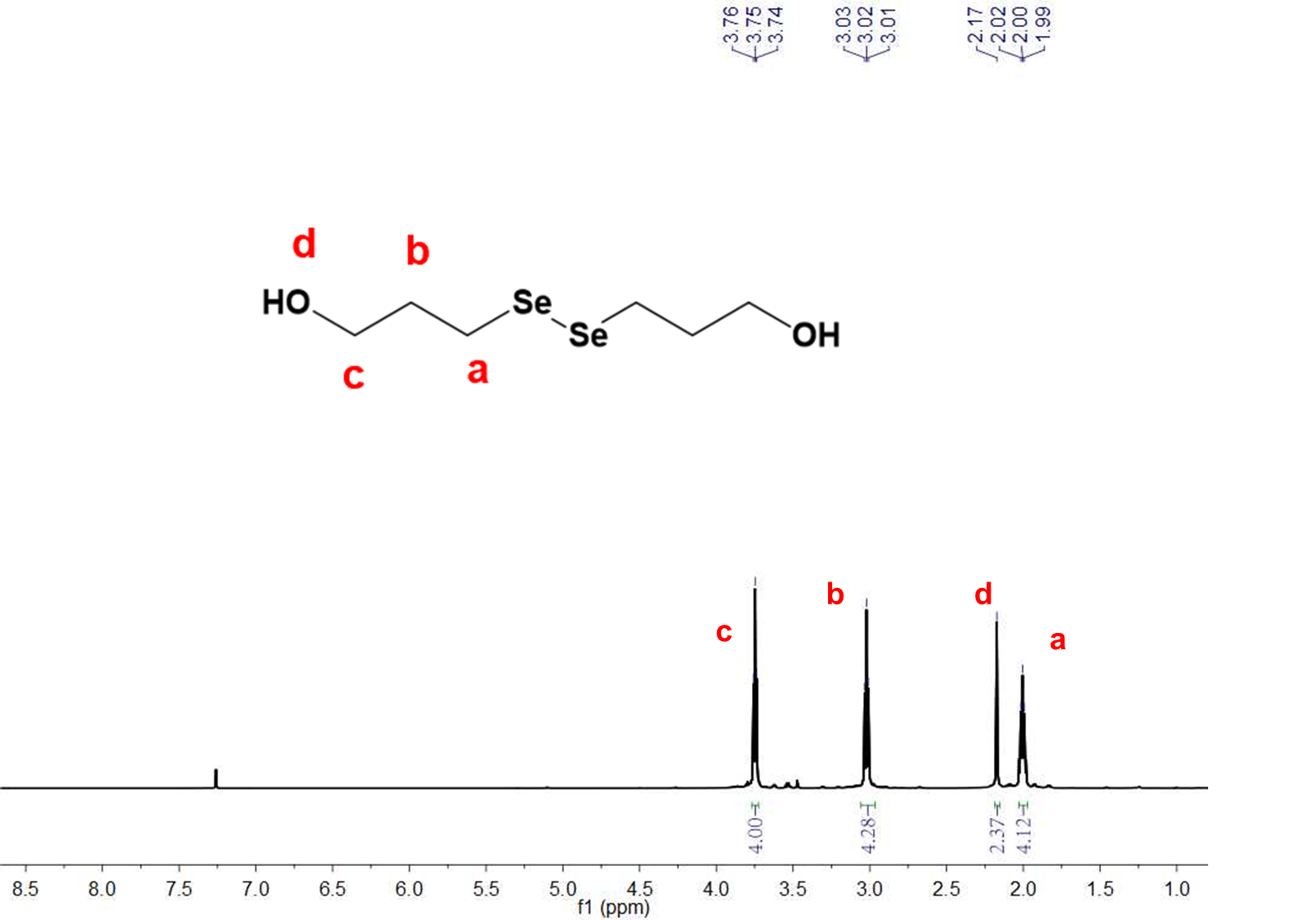


**Figure S2.** ^1^H NMR spectrum of compound **1**.


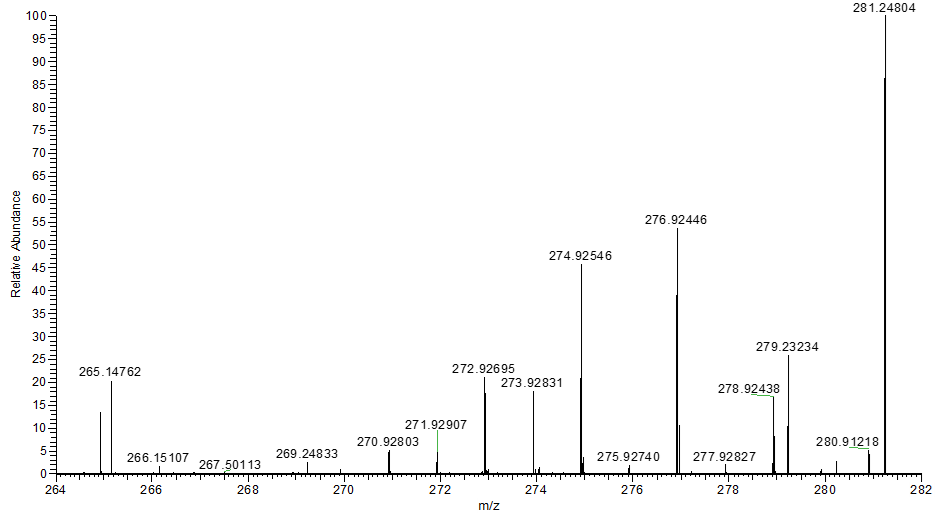


**Figure S3.**  High-resolution mass spectrum of compound **1**.

Compound **1** (4.61 g, 16.67 mmol), triethylamine (3.60 g, 39.98 mmol) and dry THF (150 mL) were added into a 250 mL Schlenk flask. Acryloyl chloride (3.61 g, 39.98 mmol) was added dropwise into the above solution under nitrogen atmosphere. The flask was sealed with a rubber stopper, and the reaction mixture was stirred at 0°C for 24 hours. The mixture was then heated to room temperature, resulting in the precipitation of salt which was removed by filtration. The filtrate was washed with a 0.1 M sodium carbonate aqueous solution. The organic layer was dried with MgSO_4_ and concentrated using a rotary evaporator. The crude oil obtained was purified by flash column chromatography using a petroleum ether and ethyl acetate mixture (petroleum ether:ethyl acetate = 20:1), yielding the desired product **OPDSPA** (4.8 g, 75% yield). ^1^H NMR (600 MHz, CDCl_3_) δ: 6.42-6.38 (dd, *J* =6 Hz, 18 Hz, 2H), 6.14-6.09 (dd, *J* =12 Hz, 18 Hz, 2H), 5.84-5.82 (d, *J* =12 Hz, 2H), 4.25-4.23 (t, *J* =6 Hz, 4H), 2.97-2.94 (t, *J* =6 Hz, 4H), 2.13-2.11 (m, 4H).


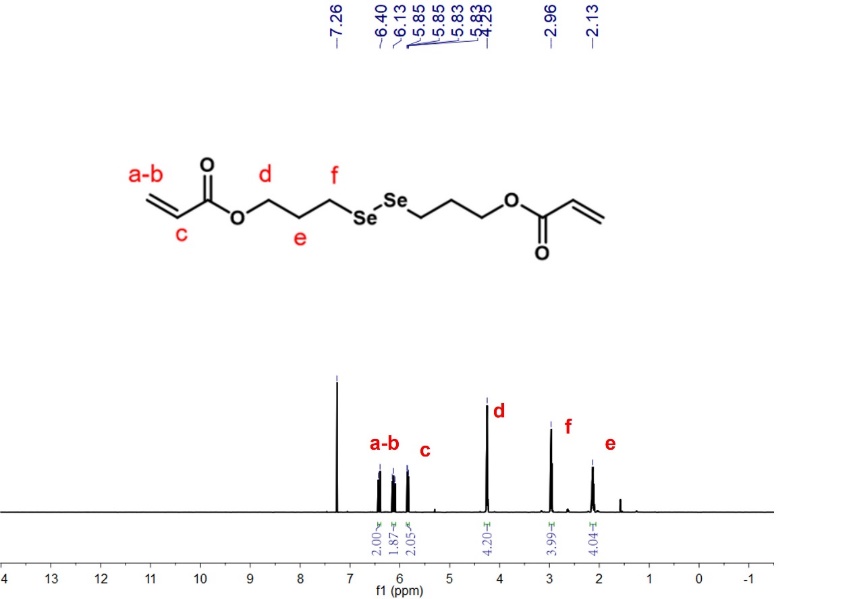


**Figure S4.** ^1^H NMR spectrum of OPDSPA.

**Preparation of** **LCE-based aerogel sample containing diselenide bonds**

The preparation protocol was divided into four main stages, including the synthesis of organogel, solvent exchange process, supercritical CO_2_ extraction and shape programming stage.


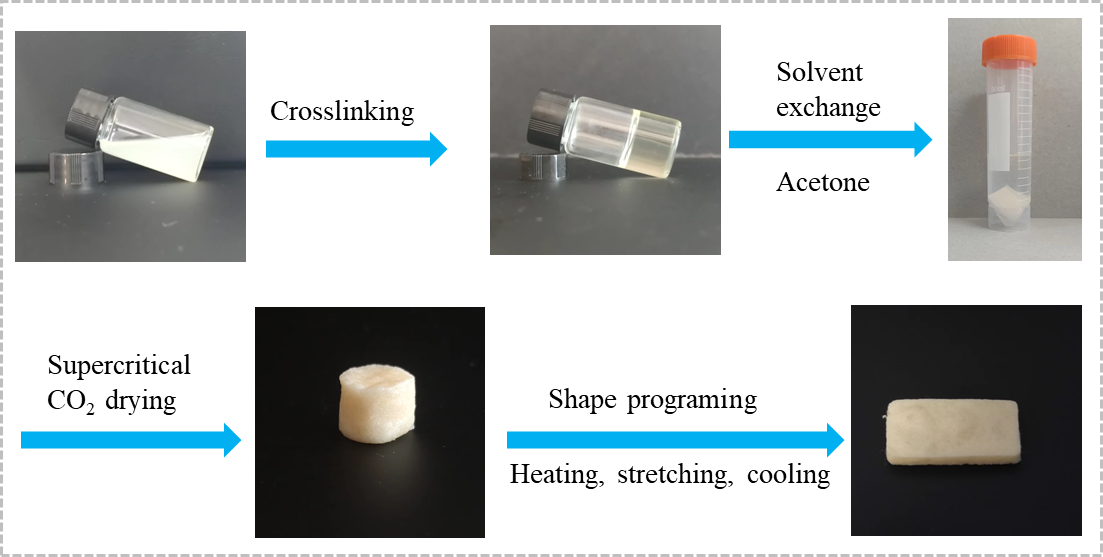


**Figure S5.** Preparation of the LCE-based aerogel.

**(1) Fabrication of organogel.** RM82 LC monomer (840 mg, 1.248 mmol), PETMP crosslinker (30.50 mg, 0.0624 mmol), DODT chain extender (230.04 mg, 1.2618 mmol), and OPDSPA (53.54 mg, 0.1388 mmol) were dissolved in 4 mL of tetrahydrofuran (THF). Subsequently, azodiisobutyronitrile (AIBN) (5 mg, 0.03 mmol) and dipropylamine (DPA) (5 μL) were added to the mixture solution. The mixture was then subjected to ultrasonication for 3 minutes. The resulting solution was carefully dripped into a 10 mL transparent glass bottle. The hermetically sealed bottle or mold containing the reaction solution was placed in an oven and heated at 50 °C for 1 hour to complete the gelation process. This resulted in the formation of a yellow, transparent cylindrical organogel.

**(2) Solvent exchange.** The resulting organogel was immersed in acetone for a duration of 72 hours, with the acetone being refreshed every 24 hours (three times in total) to ensure thorough removal of residual THF and impurities from the organogel. As a result of this solvent exchange process, the initially transparent organogel underwent a transformation, becoming opaque and acquiring a milky white appearance.

**(3) Supercritical CO_2_ extraction.** The organogel soaked in acetone was further treated by exchanging the acetone with liquid CO_2_ using an Accudyne multivessel automated system. Firstly, wet organogel with the bottle was placed into a sealed separation kettle. After turning the inside temperature to 25 °C, the separation kettle was pressurized to 10.0 MPa with CO_2_ pump. Then, the acetone in the bottle was continuously exchanged by liquid CO_2_ at the flow rate of 15 L/min. This solvent replacement process was repeated at least three times until no liquid flowed out from the collection vessel. After that, keeping the pressure in stable, the inside temperature of the separation kettle was raised to 50 °C. The gel was constantly washed with supercritical CO_2_ at the flow rate of 10 L/min. This procedure lasted for 4-6 h to finish the whole CO_2_ extraction process. Finally, the CO_2_ in the separation kettle was gradually released at a degassing rate lower than 5 L/min until the pressure in the kettle was equal to the atmospheric pressure. The samples were placed in a vacuum oven for 12 h to remove any solvent residue.

**(4) Shape programming.** The sample was cut into strips and annealed at 120 ^o^C for 40 minutes to induce diselenide bond cleavage. Then, the sample was stretched to 250% of the original length, fixed, cooled to room temperature, and left undisturbed for 24 hours to obtain the monodomain LCE-based aerogel sample.


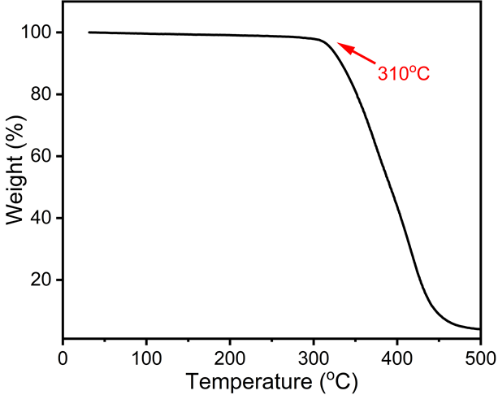


**Figure S6.** TGA curve of LCE-based aerogel sample at a heating rate of 10.0 ^o^C /min under nitrogen atmosphere.

**
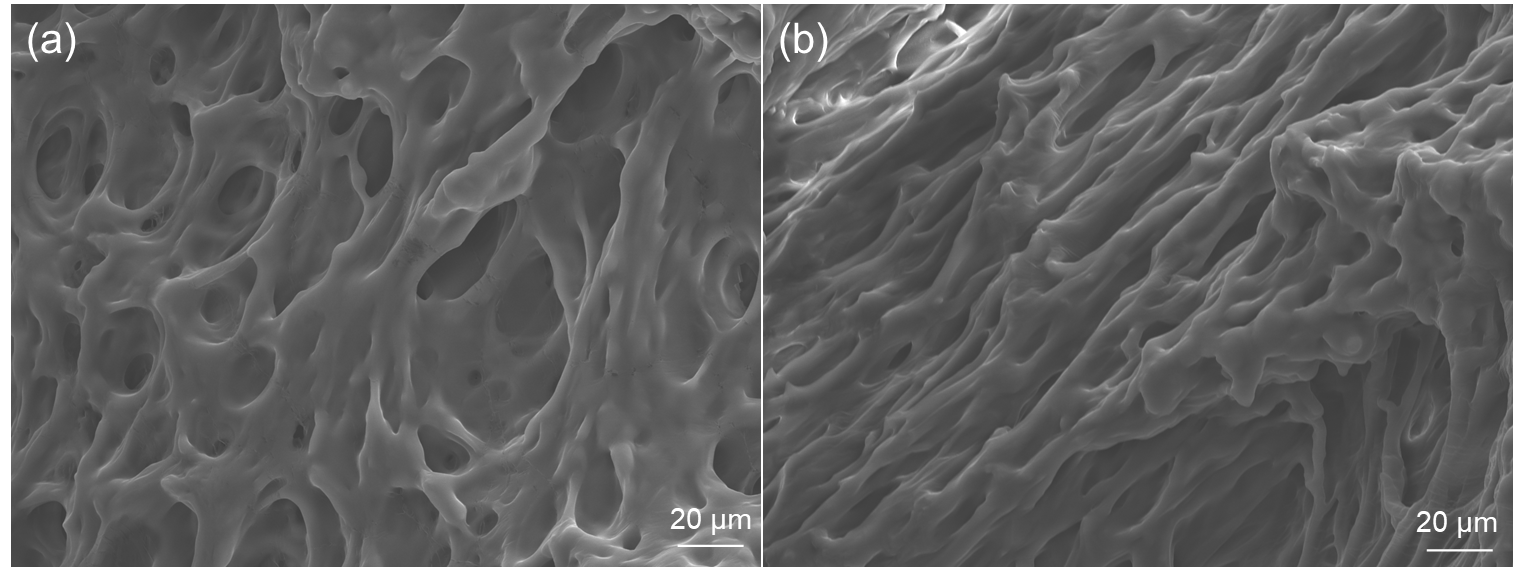
**

**Figure S7**. SEM images of the LCE-based aerogel sample (a) before and (b) after strectching.

**Table S1.** Contents of four elements on the surface of LCE-based aerogel samples

| Chemical element | C | O | S | Se |
| --- | --- | --- | --- | --- |
| Wt % | 70.35 | 19.74 | 8.37 | 1.54 |

**
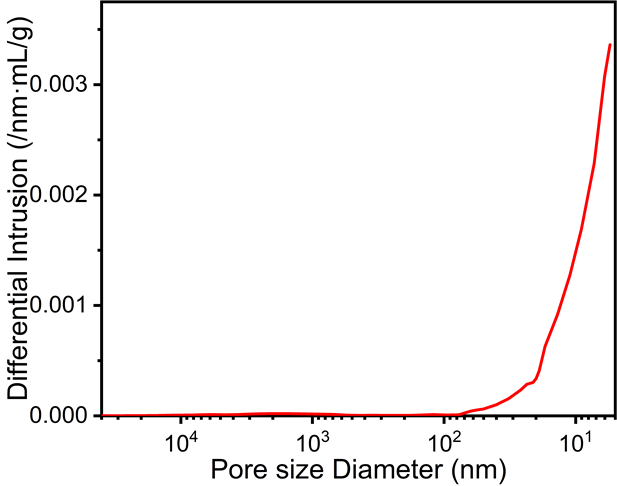
**

**Figure S8.** Curve of differential invasion versus pore size of LCE-based aerogel sample in MIP test.

**
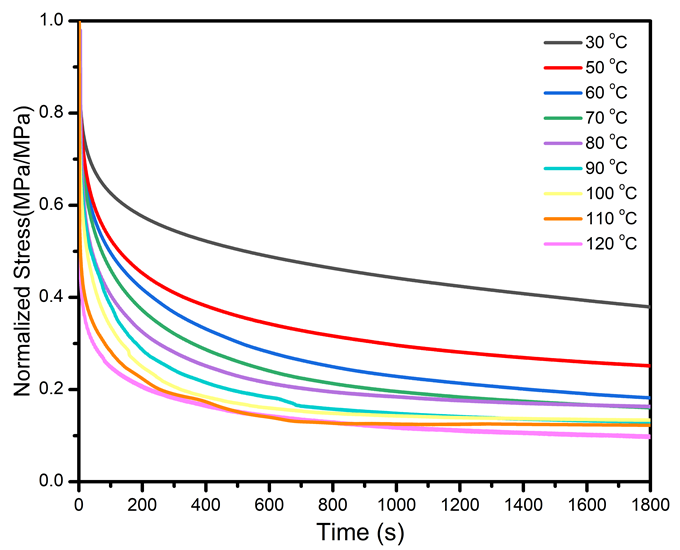
**

**Figure S9.** Normalized stress relaxation curves of LCE-based aerogel samples at different temperatures ranging from 30 °C to 120 °C.

**
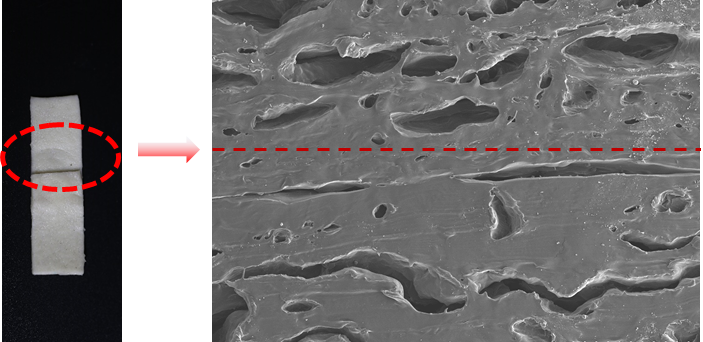
**

**Figure S10.** SEM picture of the interface between two overlapped LCE-based aerogel samples.

**
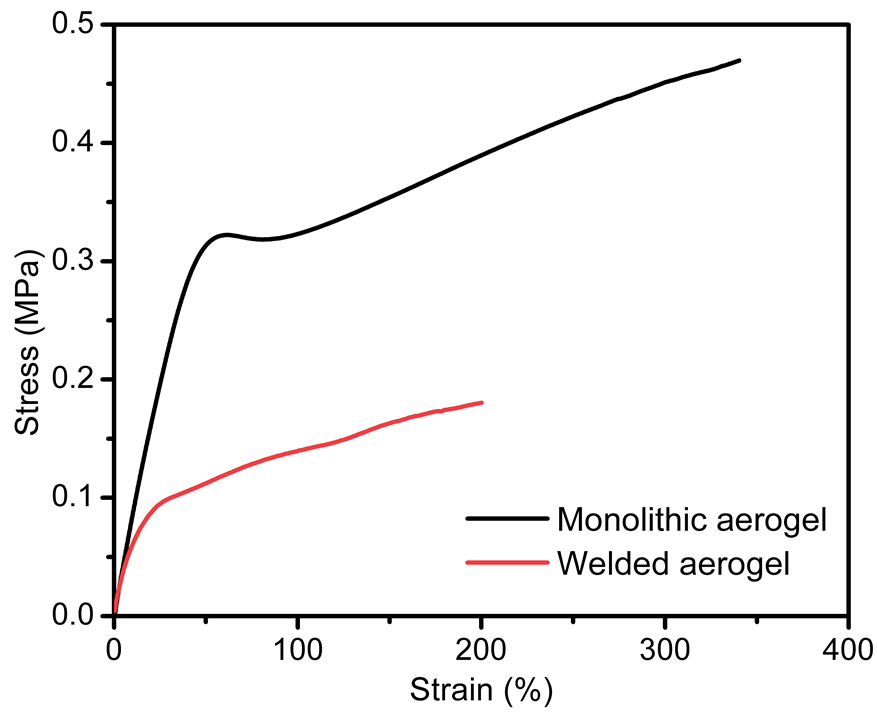
**

**Figure S11** Tensile stress-strain curves of (a) monolithic and (b) welded LCE-based aerogel samples.

**
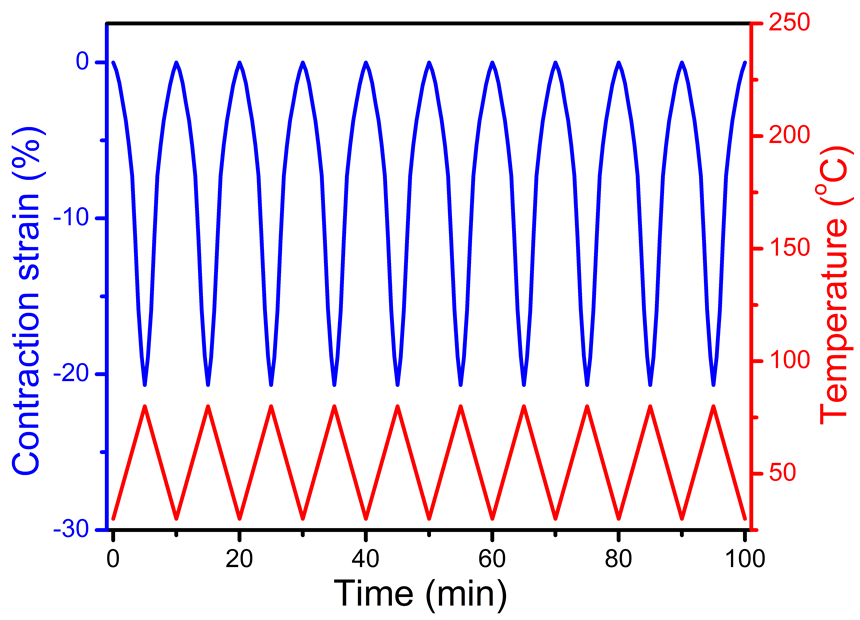
**

**Figure S12.** Representative strain (in isoforce mode) and the corresponding temperature diagram of the LCE-based aerogel sample plotted against time.


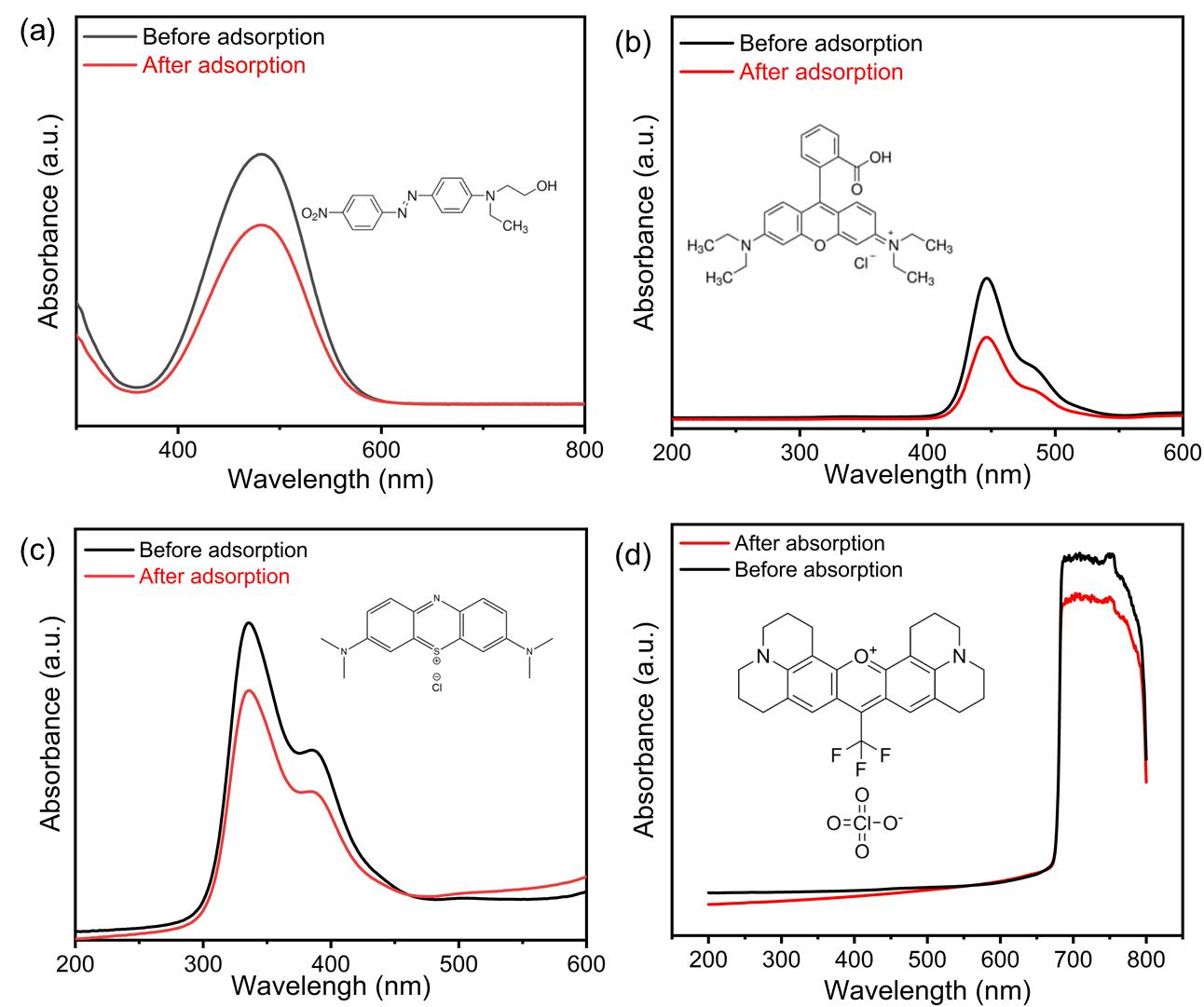


**Figure S13.** UV-vis absorption spectra of different composite solutions: (a) DR1/ethanol (10 mg/L), (b) Rhodamine B/ethanol (10 mg/L), (c) methylene blue/ethanol (10 mg/L), (d) Rhodamine 700/ethanol (10 mg/L).


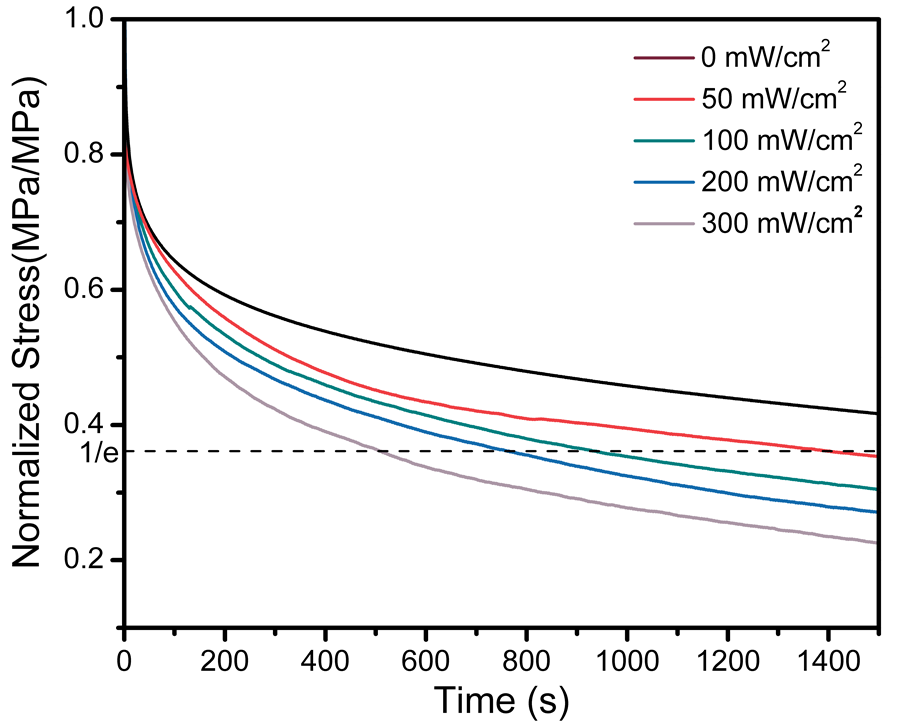


**Figure S14.** Normalized stress relaxation curves of LCE-based aerogel samples under 520 nm light with different intensities ranging from 0 to 300 mW/cm^2^.


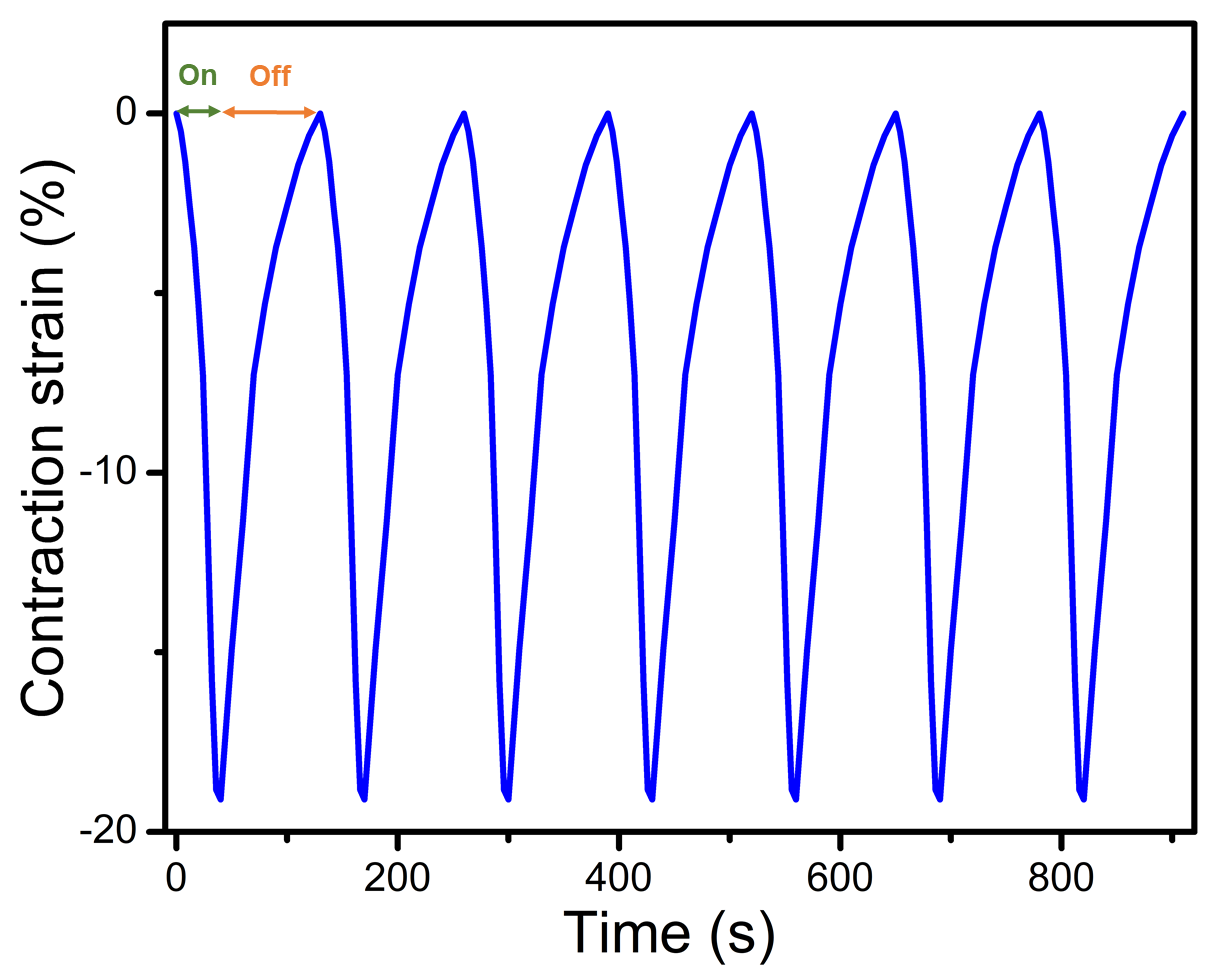


**Figure S15.** Representative strain (in isoforce mode) diagram of the DR1/LCE-based aerogel sample plotted against time during the cycles of green light exposure.
